# The lateral joint line opening: a radiographic indicative parameter for high grade varus knees

**DOI:** 10.1186/s40634-022-00489-5

**Published:** 2022-05-30

**Authors:** William Colyn, A. Cleymans, L. Bruckers, R. Houben, K. Smeets, J. Bellemans

**Affiliations:** 1 Department of Orthopedic Surgery, AZ Turnhout, Steenweg Op Merksplas 44, 2300 Turnhout, Belgium; 2 Faculty of Medicine and Life Sciences, Hasselt University, Diepenbeek, Belgium; 3 Ziekenhuis Oost-Limburg, Dept. Future Health, Genk, Belgium; 4 Department of Orthopedic Surgery, University Hospitals Leuven, Leuven, Belgium; 5 I-BioStat, University Hasselt, Hasselt, Belgium; 6 Deparment of Orthopedic Surgery, AZ Vesalius, Tongeren, Belgium; 7 Faculty of Rehabilitation Science, Hasselt University, Diepenbeek, Belgium; 8 GRIT Belgian Sports Clinic, Leuven, Belgium; 9 Department of Orthopedic Surgery, ZOL Genk, Genk, Belgium

## Abstract

**Purpose:**

It is usually assumed that the severity of varus osteoarthritis (OA) of the knee is correlated with the axis deviation of the limb. Despite this, there is currently no clear radiographic definition to define a so-called ‘high degree’ varus knee, which is characterized by a pronounced lateral ligamentous laxity. The purpose of this study was to radiographically determine if the lateral joint line opening (LJLO) is an indicative parameter when defining so-called high grade varus knees.

**Methods:**

Two hundred forty Full length radiographs of patients with end-stage varus osteoarthritis who were scheduled for Total knee arthroplasty (TKA) were evaluated. The Hip-knee-ankle-angle (HKA-angle), Joint-line-convergence-angle (JLCA) and the lateral joint line opening were measured. The lateral joint line opening is the shortest distance between the lateral tibial plateau and the deepest point of the lateral femoral condyle. Linear regression models were used to investigate the relationships between the radiographic measurements.

**Results:**

Hip-knee-angle-angle, joint-line-conversion-angle, and lateral joint line opening were all positively correlated (*p* < 0.001). An increase of 1 mm lateral joint line opening causes an increase of 0.6° joint-line-conversion-angle (*p* = 0.029) below a cut-off point of 4.7 mm. For lateral opening values beyond 4.7 mm, the gradient increased to 1.2 (*p* < 0.001). A lateral joint line opening of 4.7 mm corresponds to a hip-knee-ankle-angle of 6.0° (95% CI [5.5; 6.5]).

**Conclusion:**

A lateral joint line opening of more than 5 mm in end-stage OA knees is indicative of increased lateral joint laxity. Those knees can be radiographically classified as so-called ‘high-grade’ varus knees.

**Level of evidence:**

Therapeutic study, Level III.

## Introduction

It is well known that the majority of knees with osteoarthritis present with a varus alignment [3, 6, 20]. In fact, about 90% of knees planned for a TKA have a varus deformity [1]. Previous research has demonstrated that the mean alignment of young healthy adults is slightly varus (1.3° )[1]. Despite the high frequency of varus knees, no clear radiographic parameter has yet been found to determine the more pronounced varus knee, a so-called a “high degree” varus knee. Previous studies made a distinction between mild (< 10°), moderate (10°-20°) and a severe (> 20°) varus deformities [10]. Thienpont et al. has recently proposed a classification, whereby a distinction is made between an intra- and extra-articular deformity [21]. The intra-articular deformities were then further subdivided: (1) reducible anteromedial osteoarthritis (AMOA) with an intact anterior cruciate ligament (ACL), (2) reducible posteromedial osteoarthritis with a deficient ACL, (3) fixed varus deformity without lateral laxity, (4) fixed varus with lateral laxity. Nevertheless, it is not clear how lateral laxity was radiographically determined. Noyes divided varus knees in three groups: (1) primary varus: narrowing of the medial tibiofemoral compartment, (2) double varus: separation of the lateral tibiofemoral compartment from moderate deficiency of the posterolateral structures which manifests as more than 5 mm increased lateral joint opening or a 10° increase in external tibial rotation and (3) triple varus: varus recurvatum in extension due to a severe deficiency of the posterolateral ligamentous structures [15]. So, this classification demonstrates a progressive medial narrowing of the joint line during the early progression of varus OA, followed by an increase of the lateral joint line opening. The progressive medial joint narrowing can be declared by the loss of medial cartilage, whereby the increase of lateral joint line can be explained by the progressive stretching of the lateral ligamentous structures [2, 16]. It is clear that during the progressive varus deformation, the strain on the lateral structures progressively increases. Provenzano quantified cellular damage in a ligament as a function of strain [19]. A damage threshold was described above which the mechanical properties of a ligament were irreversibly changed. Therefore, it was hypothesized that during the progression of varus osteoarthritis a certain threshold exists, after which progressive lateral laxity appears. As there is a threshold after which progressive lateral joint laxity appears, we are convinced that it can be radiographically determined. Until now, there is no straightforward radiographic parameter to determine this progressive lateral knee joint laxity on a standard standing X-ray. The aim of this study is to radiographically quantify if the lateral joint line opening (LJLO) is an indicative parameter when defining the so-called high grade varus knees. It is expected to find a threshold value for the LJLO after which the lateral knee joint laxity progresses.

## Methods

The radiographic databases of AZ Turnhout and Zol Genk were used. All patients who had undergone a TKA for end stage degenerative osteoarthritis between July 2016 and August 2019 were evaluated. Two orthopedic surgeons (WC, JB) performed the surgeries. Inclusion criteria were knees with preoperative grade 3 or 4 Kellgren-Lawrence tibiofemoral osteoarthritis, patients aged at least 50 and an HKA-angle of more than 0° varus. Full length radiographs were obtained with the patellae facing forward [14]. Patients with an ipsilateral total hip prosthesis (THP), preoperative femoral or tibial fracture, osteotomy or ligamentous repair were excluded.

All radiographs were evaluated for image quality and good rotational positioning. An appropriate positioning was defined by the patella facing forward; a similar shape of the lesser trochanters; and a similar overlap of the proximal tibiofibular joint.

The radiographic measurements were performed by one observer (C) using the AGFA PACS software package (Agfa-Gevaert), which produced a measurement accuracy of up to 0,1°. Inter-observer reliability was determined by intra-class correlation coefficients (ICCs) for the angle measurements of 100 knees, performed by a second observer (D). In total, 3 alignment parameters were measured using the previously described techniques.

**Fig. 1:**
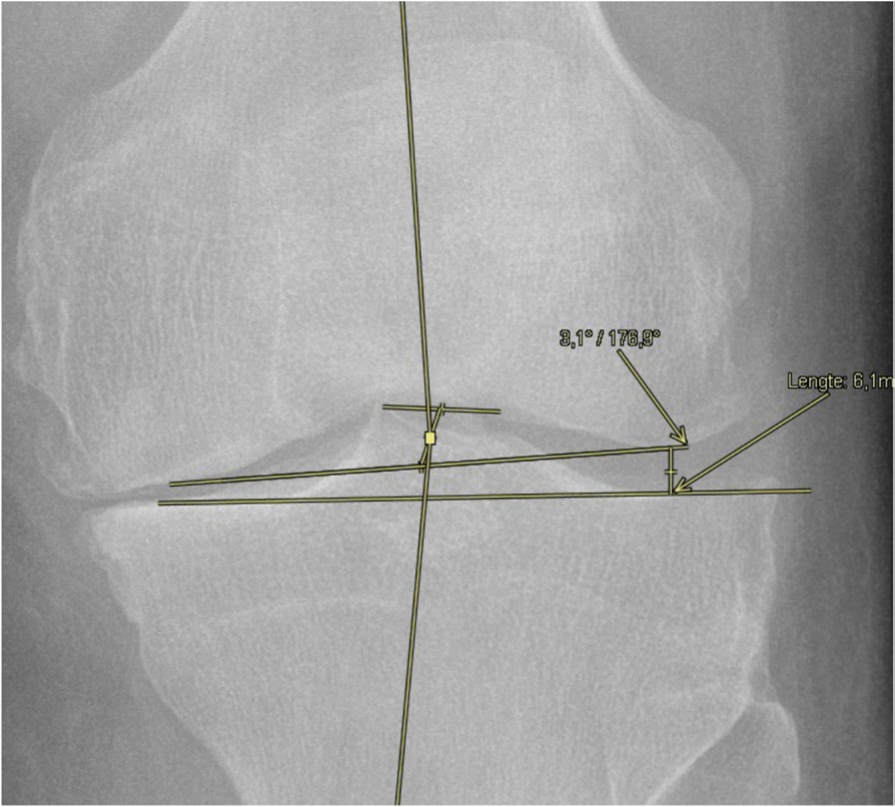
The Lateral joint line opening (LJLO) is measured as the distance between the lateral tibial plateau and the lateral femoral condyle. The LJLO in this illustrative figure measures 6,1 mm

1. The HKA angle was formed by the mechanical femoral axis and the mechanical tibial axis. The center of the hip was defined by the center of a corresponding concentric circle. The center of the knee was defined by the intersection of the midline between the tibial spines and the midline between the most distal points of the lateral and medial femoral condyles. The center of the ankle was defined as the middle of the proximal surface of the talus [17].
2. The joint line convergence angle (JLCA) was formed between the knee joint lines of the distal femur and the proximal tibia [17].
3. The lateral opening was measured as the shortest distance between the lateral tibial plateau and the intersection of the joint line and the deepest point of the lateral femoral condyle (Fig. 1).

The degree of arthrosis was determined based on the Kellgren-Lawrence scale (KL-scale) [12].

### Statistics

Linear regression models were used to investigate the relationships between the radiographic measurements. To improve the model fit, piecewise linear regression models were also utilized. The relationship between the dependent variable and the independent variable was assumed to consist of 2 linear segments. The breaking point was estimated based on the data.

## Results

Overall, 362 patients had radiographs that met the inclusion criteria based on alignment, age and arthritic disease. In total, 122 radiographs were excluded: 48 due to poor quality related to patient positioning, 17 due to the presence of a prior femoral or tibial fracture, 6 due to a pre-operative osteotomy, 19 due to prior ligamentous repair, 32 due to an ipsilateral hip prosthesis. The final analysis included 240 radiographs (135 men and 105 women) with an age range of 47 to 90 years (mean: 72 years). All measurements demonstrated a good accuracy with ICCs for inter-observer reliability: 0,97 (HKA); 0,75 (JLCA) and 0,69(LJLO).

**Fig. 2:**
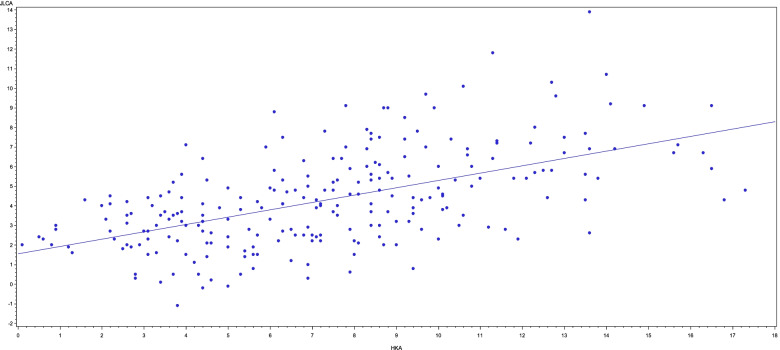
In this graph, the HKA angle is seen on the x-axis. Plotted against it on the Y-axis is the JLCA, also measured in degrees. A positive linear correlation is observed. The *p*-value for this correlation is 0.001

The mean HKA was 7.5°. Our results showed a significant correlation between HKA and JLCA (*p* = 0.001). The slope of this curve is 0.4 whereby an increase of 1° in HKA is associated with an increase in JLCA of 0.4° (Fig. 2).

**Fig. 3:**
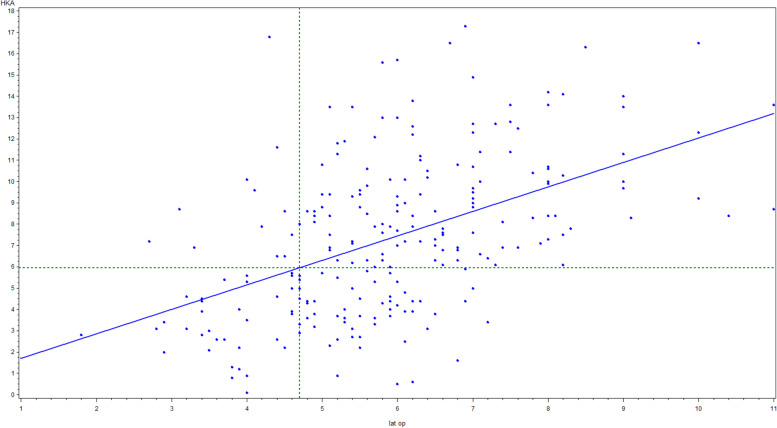
In this graph, the lateral opening (measured in mm) is plotted on the X-axis and the HKA (measured in degrees) on the Y-axis. The corresponding mean HKA for a lateral opening of 4.7 mm is 5.95 with a 95% CI [5.45; 6.45]

A positive correlation was demonstrated between the lateral opening and the HKA. The gradient of the curve was calculated to be 1.2 (*P*-value < 0,001) (Fig. 3).

**Fig. 4:**
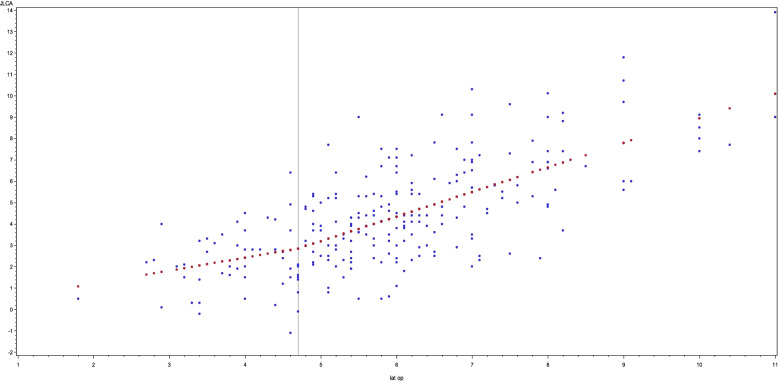
In this graph, the lateral opening (measured in mm) is plotted against the JLCA (measured in degrees). A positive correlation is found between the two variables. However, a difference in gradient is clearly seen with the cut-off point being 4.7 mm. The gradient beyond this point is almost double that compared to the gradient before this point

In addition, a positive correlation was found between the lateral joint line opening and the JLCA (*p* < 0.001). A clear change in gradient was found at 4.7 mm of lateral joint line opening. For lateral opening values below this point, the gradient was 0.6 (*p* = 0.03) and above this point, the gradient was 1.2 (*p* = 0.09) (Fig. 4).

## Discussion

The most important finding of this study was that knees with a lateral joint line opening ≥5 mm are associated with an increasing lateral joint laxity. The lateral joint line opening is a very simple radiographic parameter used to define the so-called high grade varus knees.

Changes in the lateral joint line opening are strongly correlated to the joint line convergence angle of the knee. The influence of the LJLO on the increase of JLCA changes remarkably after the cut-off point of 4.7 mm. An increase of 1 mm LJLO below the threshold-value of 4.7 mm increases the JLCA by 0.6° (*p* < 0.05). Above the cut-off point, this increase amounts to 1.15° (*p* < 0.001); the slope beyond this point therefore almost doubles. In other words, the contribution of LJLO to the change in JLCA is rather limited below a lateral opening of 4.7 mm however, above this cut-off point it has a more pronounced effect.

A greater JLCA is also correlated with a greater varus deformity (*p* = 0.001). Logically, progression in HKA is influenced by the changes in the distal femur, the proximal tibia and the joint line convergence angle: HKA = MPTA + LDFA + JLCA [8]. The JLCA is determined by the gaps between the tibial and femoral joint lines. This can be due solely to cartilage wear and joint space opening. In varus OA knees, the cartilage volume diminishes medially, whereby laterally, the joint laxity will increase over time [13].

During the first stage of the development of varus OA knees, the ground reaction force results in an increased stress on the medial side of the knee, resulting in medial cartilage wear [3, 4]. As a consequence, the JLCA will increase. Another factor known to change the JLCA is a progressive stretching of the lateral complex, due to the adduction moment pushing the knee outward [22]. This can be interpreted as repeated microtrauma. Such ligament microtrauma may result in increased laxity [9]. Early studies of mechanical properties of ligaments showed that irreversible mechanical changes occur at relatively low levels of strain [19]. Previous studies suggested that the shape of loading curves is altered after reaching a sub failure injury [19]. Thereby, the joint laxity increases [18]. Structural and cellular damage occurs in the ligaments after reaching the threshold level of strain [19]. Such damaged ligaments are characterized by a nonrecoverable change in tissue length [19]. The resulting increase in tissue length represents a tissue laxity and can be hypothesized to increase joint laxity. The radiographic findings from this study suggest that 4.7 mm lateral joint line opening is the cutoff from when the joint laxity increases. In those knees, a structural and cellular changes in the lateral ligamentous structures are likely to occur. Those are the so-called ‘high-degree’ varus knees. These findings are therefore in line with the Noyes classification, whereby a 5 mm increased lateral joint line opening was one criterion for a ‘double varus knee’ [15]. The 4.7 mm lateral joint line opening correlates with an HKA of 5.95° (Fig. 2).

There are several limitations in this study. Firstly, this study depended on a single observer setting; the measurements may have been influenced by the accuracy of the investigator, although a single observer guarantees consistency. Secondly, rotational malalignments of a full-length radiograph are possible and could have interfered with our measurements. The full leg radiograph has however been described as a useful tool for quantification of different measurements with an outstanding intra- and interobserver reliability [7, 8]. Moreover, radiographs with a rotational malposition were excluded. The inclusion criteria were a forward-facing patella, a similar shape of the lesser trochanters; and a similar overlap of the proximal tibiofibular joint [5, 10, 11]. Thirdly, full leg radiographs were acquired from end stage OA patients, scheduled for TKA. No sequential X-rays of those patients were investigated. So, evolution within subjects cannot be fully excluded. Fourthly, only radiographic measurements were used. Obviously, the addition of clinical investigations or histological and biomechanical studies of the lateral ligamentous complex of end-stage OA knees could provide added value in future research projects.

Based on these findings, it is suggested that the lateral joint line opening is measured on pre-operative X-rays. If the LJLO is ≥5 mm, an insufficient lateral complex could be expected. Knee surgeons should be mindful of medio-lateral disbalance, when performing total knee arthroplasty in such knees. An inadequate release or an outstretched lateral complex may result in a ML instability. The use of more constrained implant designs could be necessary to compensate for this medio-lateral disbalance in such high-grade varus OA knees.

## Conclusion

A radiographic parameter is defined to determine the so-called high-grade varus knees. Those knees, with a lateral joint line opening of more than 4.7 mm are characterized by a progressive outstretched lateral complex. Performing TKA in such knees may result in an imperfect soft tissue balance.
